# Scalable synthesis of N,S co-doped honeycomb-like porous carbon with micropore-dominance for ultrahigh volumetric-performance supercapacitors

**DOI:** 10.1039/d5ra08165c

**Published:** 2026-01-12

**Authors:** Chong Chen, Yongxiang Su, Wenxiu Zhong, Keying Zhang, Pinghua Zhang

**Affiliations:** a Key Laboratory of Spin Electron and Nanomaterials of Anhui Higher Education Institutes, School of Chemistry and Chemical Engineering, Suzhou University Suzhou 234000 People's Republic of China szxychenchong@163.com zphchemical@163.com

## Abstract

The conversion of low-cost, sustainable biomass into high-value porous carbon as an electrode material for supercapacitors has attracted considerable recent attention. Almost all efforts are focused on developing advanced carbon electrode materials without conducting large-scale experiments. Utilizing cotton pulp board as the prototype, we demonstrate that honeycomb-like porous carbon could be completely and repeatedly obtained by a one-step activation method. The preparation method is facile and industrially feasible, and could be used for the scalable production of multiple heteroatom-doped honeycomb-like porous carbon (MHDHPC) with excellent reproducibility and high yield. MHDHPC exhibits ultrahigh gravimetric capacitance and volumetric capacitance due to its high surface area, ultrahigh microporosity, relatively high density and surface heteroatom-rich nature. A MHDHPC//MHDHPC based symmetric supercapacitor can deliver a superior volumetric energy density of 13.1 Wh L^−1^ in 6 M KOH. These exciting results provide a sustainable, scalable and low-cost method to prepare MHDHPC for high volumetric-performance supercapacitors.

## Abstract 2

The conversion of low-cost, sustainable biomass into high-value porous carbon as an electrode material for supercapacitors has attracted considerable recent attention.

## Graphical abstract

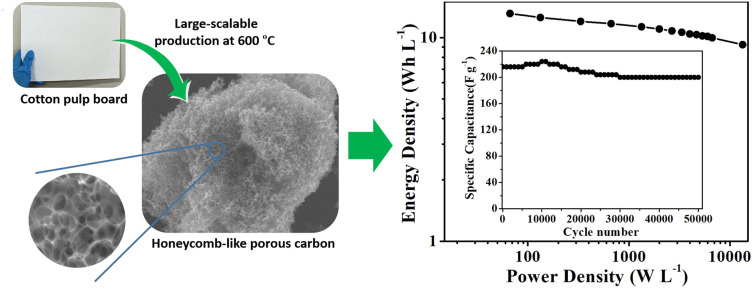

## Introduction

The growing depletion of fossil fuel resources and the escalating environmental pollution associated with their use present serious challenges. To address these problems, great progress has been made in the exploitation and utilization of sustainable energy and efficient energy storage devices. Carbon-based supercapacitors (CSCs) have been developed rapidly in the past decades, due to their high-power density, long cycle life, abundant and renewable carbon-based electrode materials, fast charging and discharging, suitability for various electrolytes (aqueous, organic and ionic liquid solutions), and a wide temperature range.^1–3^ The development of economical and efficient carbon-based electrodes is crucial for CSCs. Advanced carbon-based electrode materials such as porous carbon, graphene, onion-like carbon, carbon nanospheres and carbon nanocages have been widely studied by researchers with the aim of improving the electrochemical performance of CSCs.^4–13^ In particular, porous carbon derived from sustainable biomass has recently attracted attention owing to its unique morphologies, high specific surface area (SSA), suitable pore distribution, tunable surface properties and low cost.^14–17^ Therefore, it is urgent to develop industrially feasible production techniques to prepare advanced porous carbon using renewable and naturally abundant resources as raw materials for CSCs.

Recently, new and advanced porous carbons with various peculiar morphologies have been prepared using biomass as raw materials, such as honeycomb-like porous carbon (HPC) and nanosheet-like porous carbon.^18,19^ Of these, HPC has attracted considerable interest because of its advantages of a highly accessible SSA, hierarchical porous structure, high pore volume, low-cost and abundant feasible biomass as raw materials. Therefore, HPC has been prepared from various biomass materials, including cauliflower,^20^ grape,^21^ lignin,^22^ shrimp shell,^23^ wheat bran,^24^ cotton seed husk,^25^*Ganoderma lucidum* spore,^26^ using activation methods. However, all previous reports only focused on the synthesis of small amounts of HPC without any large-scale experiments. Moreover, these HPC materials generally exhibit low volumetric capacitance and low volumetric energy density due to their low density. For example, HPC derived from garden cress seeds exhibits a relatively low volumetric energy density.^27^ To obtain porous carbon with high volumetric performance, much effort has been devoted to preparing porous carbon with a high density, low pore volume and high SSA. For example, HPC with a high density (1.02 g cm^−3^), low pore volume (0.48 cm^3^ g^−1^) and high SSA (818 m^2^ g^−1^) exhibits an ultrahigh volumetric capacitance of 502 F cm^−3^.^28^ Some biomass materials like fungus,^29^ soybeans,^30^ pomelo peel,^31^ elm samara,^32^ and jujube^33^ have been demonstrated to be efficient precursors for preparing porous carbons with high volumetric performances, although they are considered food, rare, or difficult to collect. Therefore, it is a great challenge to obtain high volumetric performance porous carbons through simple, highly scalable, highly available, and cost-effective synthesis methods for CSCs.

The introduction of nitrogen and sulfur into the carbon framework is an effective method to improve the electrochemical performance of supercapacitors in aqueous electrolytes, through improving the wettability of carbon materials and generating pseudocapacitance *via* redox reactions.^34–37^ N,S co-doped porous carbons have been intensively studied in supercapacitors, but some expensive and toxic N and S reagents (*e.g.* thiourea) are generally required in their preparation process. In our previous works, we have demonstrated that N,S co-doped porous carbons could be prepared from biomass materials (*e.g.* instant dry yeast,^28^ ant powder,^38^ and bean worm^39^) *via* a one-step activation method without adding any N and S reagents, although this method suffered from low yields. It would be preferable to prepare N,S co-doped porous carbons using an inexpensive and heteroatom-rich biomass precursor. Cotton pulp board produced from natural cotton linters and waste cotton in the textile industry is generally composed of pure cellulose. Considering the combination of its low cost, worldwide abundance, heteroatom-rich nature and ready availability as an industrial product, cotton pulp has been used as a carbon precursor for the production of carbon fibre materials.^40–44^ Although some progress has been made, developing a one-step strategy to synthesize N,S co-doped porous carbon directly from cotton pulp, without adding any N and S reagents, remains desirable.

Herein, we report a one-step activation method for the scalable production of multiple heteroatom-doped honeycomb-like porous carbon (MHDHPC) by using cotton pulp board without adding any N and S reagents. Cotton pulp board is purchased from Anhui Snow Dragon Fibre Technology Co., Ltd for this work. Cotton pulp board contains abundant heteroatoms, including N (8.8%), O (23.6%), and S (0.6%) (Fig. S1 and Table S1), which should lead to better preparation of N,S co-doped porous carbons. The N and S doping in cotton pulp board could be attributed to the usage of chemical reagents such as *N*,*N*-dimethylformamide and sodium hydroxyethyl sulfonate in the manufacturing processes. In addition, the uniform chemical composition and structure make cotton pulp board very suitable for the preparation of porous carbon with good reproducibility. MHDHPC has microporous features with a high SSA, high density and surface heteroatom-rich nature, and exhibits outstanding gravimetric and volumetric electrochemical performances in 6 M KOH. MHDHPC exhibits a higher yield, better reproducibility and superior volumetric electrochemical performance compared with other biomass-derived N,S co-doped porous carbon obtained by the one-step activation method,^28,38,39^ although some biomass materials are considered luxury, rare, or difficult to collect. More importantly, we demonstrate that MHDHPC shows excellent reproducibility after sixteenfold amplification experiments.

## Experimental

Cotton pulp board was purchased from Anhui Snow Dragon Fibre Technology Co., Ltd without further purification. 1.26 g of cotton pulp board was cut into a square shape with length, width and thickness of about 50 mm, 50 mm and 10 mm, respectively. The square cotton pulp board was placed in a glass dish, and then 10.0 mL of potassium hydroxide (KOH, Aladdin, 1.26 g) aqueous solution was added dropwise. The mixture was first dried at 100 °C for 6 h in a drying oven, and then was carbonized at 600 °C for 2 h in a tube furnace with a heating rate of 5 °C min^−1^. The obtained black material was washed by 1 M HCl solution for 24 h, followed by filtration with distilled water, and then dried at 100 °C for 12 h to obtain 0.172 g of MHDHPC-600. MHDHPC-600-100 and MHDHPC-600-400 were obtained when the square areas of the cotton pulp were 100 cm^2^ and 400 cm^2^.

For comparison, MHDHPC-500 and MHDHPC-700 were produced at 500 °C and 700 °C, respectively, with the same preparation process as for MHDHPC-600. To prepare other comparative samples, the cotton pulp board was mixed with different amounts of KOH with the same preparation process as for MHDHPC-600, where *n* (0.5 and 1.5) is the mass ratio of KOH : cotton pulp board. The resultant comparative samples were denoted as KOH–CP*n* (*n* = 0.5, 1.5).

Details of the characterization and electrochemical measurements of MHDHPC-500, MHDHPC-600, MHDHPC-700, MHDHPC-600-100 and MHDHPC-600-400 are given in the SI.

## Results and discussion

**Fig. 1:**
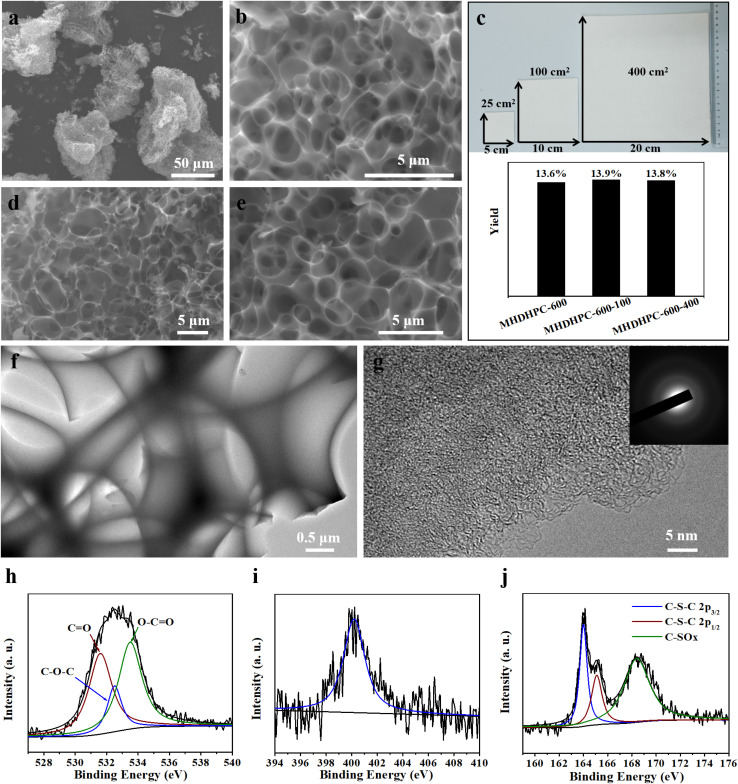
(a and b) SEM images of MHDHPC-600; (c) yield of MHDHPC-600, MHDHPC-600-100 and MHDHPC-600-400; high-magnification SEM images of (d) MHDHPC-600-100 and (e) MHDHPC-600-400; (f and g) TEM images of MHDHPC-600; (h) O 1s, (i) N 1s and (j) S 2p spectra of MHDHPC-600.

MHDHPC was prepared by a one-step activation method using cotton pulp board as a precursor and KOH as a chemical activator. Fig. 1a shows the morphology of MHDHPC-600, displaying an overall honeycomb-like porous structure. A close-up SEM image (Fig. 1b) exhibits the smooth surface and three-dimensional interconnected macroporous network nature of MHDHPC-600. The honeycomb-like interconnected macroporous network structure (Fig. S2c–f) is well retained when the activation temperature is 500 °C and 700 °C. We carried out scalable production experiments in which the square area of cotton pulp board was increased from 25 cm^2^ to 400 cm^2^. Importantly, the yield (Fig. 1c) of MHDHPC-600 is 13.6%, which is almost identical to those of MHDHPC-600-100 (13.9%) and MHDHPC-600-400 (13.8%), indicating the very high stable productivity of MHDHPC. The yield of MHDHPC-600 is higher than that of MHDHPC-700 (8%) and is slightly lower than that of MHDHPC-500 (15.6%). Both MHDHPC-600-100 (Fig. 1d and S2a) and MHDHPC-600-400 (Fig. 1e and S2b) exhibit the same morphological structure as MHDHPC-600, demonstrating excellent reproducibility and reliability of MHDHPC. The same morphological structure of MHDHPC-600, MHDHPC-600-100 and MHDHPC-600-400 could be due to the uniform fibre-like structure and pure cellulose components of the cotton pulp board – the cotton pulp board was a standardized industrial product. The TEM image (Fig. 1f) of MHDHPC-600 further confirms the three-dimensional interconnected macroporous structure, in which the bright feature corresponding to the macroporous wall and connecting regions illustrates the very thin overall macroporous network. The high-resolution TEM image (Fig. 1g) exhibits the highly microporous features of MHDHPC-600, and the inserted selected-area diffraction pattern reveals a highly amorphous and disordered structure in MHDHPC-600. The XPS survey spectrum (Fig. S3a) of MHDHPC-600 shows clearly a narrow graphitic C 1s peak (87.8%) and O 1s peak (8.8%), as well as an inconspicuous N 1s peak (2.1%) and S 2p peak (1.3%), indicating that abundant multiple heteroatoms survive after the strong activation process. These heteroatoms are derived from cotton pulp board containing various heteroatoms: N (8.8%), O (23.6%), and S (0.6%). In sharp contrast, the XPS survey spectrum (Fig. S4a) of MHDHPC-700 contains C (88.6 at%), O (10.4 at%) and N (1.3 at%), but S is absent (Fig. S4b), indicating that the S atom has been completely etched by an excessive KOH activation effect. Both MHDHPC-600-100 and MHDHPC-600-400 are also composed of C, O, N and S elements, but have lower contents of N and S than MHDHPC-600 (Table S1). The high-resolution C 1s spectra (Fig. S3b) of MHDHPC-600, MHDHPC-600-100 and MHDHPC-600-400 are resolved into five individual peaks, corresponding to C–S (283.8 eV), C–C (284.8 eV), C–N (285.6 eV), C=O (286.5 eV), and O–C=O (288.8 eV) binding.^37^ The O 1s XPS spectra (Fig. 1h and S3c) of MHDHPC-600, MHDHPC-600-100 and MHDHPC-600-400 can be deconvoluted into three peaks: C=O (531.6 eV), C–O–C (532.5 eV), and O–C=O (533.5 eV). The N 1s XPS spectra of MHDHPC-600, MHDHPC-600-100 and MHDHPC-600-400 (Fig. 1i and S3d) could be fitted to a single peak centred at 400.2 eV corresponding to pyrrolic N. The S 2p XPS spectra of MHDHPC-600, MHDHPC-600-100 and MHDHPC-600-400 (Fig. 1j and S3e) present three dominant peaks located at 164.0, 165.1 and 168.4 eV, corresponding to two sulfur species in C–S bonding and C–SO_*x*_, respectively. These nitrogen, oxygen and sulfur species could improve the wettability of MHDHPC samples and contribute to the generation of pseudocapacitive capacitance.

**Fig. 2:**
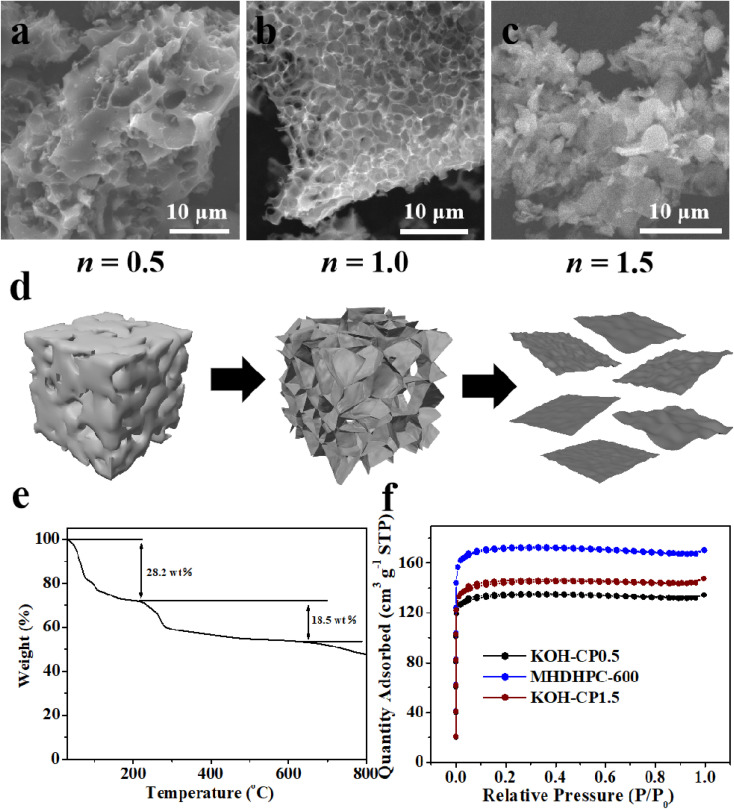
SEM images of (a) KOH–CP0.5, (b) MHDHPC-600 and (c) KOH–CP1.5; (d) schematic illustration showing the formation mechanism of MHDHPC; (e) TGA data of KOH and the CP mixture with a mass ratio of 1 : 1; (f) N_2_ sorption isotherms of KOH–CP0.5, MHDHPC-600 and KOH–CP1.5.

Direct carbonization of cotton pulp board gives rise to a fibre-like carbon material (Fig. S5), and the obtained sample is named KOH–CP0. The key to realizing honeycomb-like porous structures is the utilization of KOH that reacts with carbon through the equation 6KOH + 2C ↔ 2K + 3H_2_ + 2K_2_CO_3_.^45^ With the addition of KOH, a highly porous structure in KOH–CP0.5 (Fig. 2a) and a honeycomb-like porous framework in MHDHPC-600 (Fig. 2b) were observed. After further increasing the amount of KOH, a sheet-like carbon material was formed in KOH–CP1.5 (Fig. 2c), due to the collapse of the honeycomb-like porous structure after deep etching (Fig. 2d). To better understand the etching behavior of KOH, the thermal decomposition behavior of the KOH–CP mixture was measured in Ar gas with a ramp rate of 10 °C min^−1^. Upon carbonization below 630 °C, two weight losses of 28.2% and 18.5% were clearly observed in the TGA curve of the KOH–CP mixture (Fig. 2e), which was very different from only one weight loss of above 90% below 450 °C for CP.^40^ The first significant weight loss ending at 220 °C, was attributed to dehydration of the CP. The second weight loss step, ending at 630 °C, was attributed to thermal cleavage of the glycosidic linkages and C–O bonds, aromatisation and activation reactions induced by KOH. The additive content of KOH affected the pore structure of KOH–CP0.5, MHDHPC-600 and KOH–CP1.5. The pore structural parameters were obtained through nitrogen physisorption measurements in liquid nitrogen (Table S2). A very sharp and steep adsorption feature below 0.1*P*/*P*_0_ was observed in the isotherms of KOH–CP0.5, MHDHPC-600 and KOH–CP1.5 (Fig. 2f), revealing the predominantly microporous structures. Pore-size distribution plots (Fig. S6) further confirm the predominantly microporous nature of KOH–CP0.5, MHDHPC-600, and KOH–CP1.5, with the majority of pores measuring below 2 nm. It should be noted that MHDHPC-600 had the highest SSA and the highest total pore volume compared to KOH–CP0.5 and KOH–CP1.5, indicating better capacitance performance in supercapacitors.

**Fig. 3:**
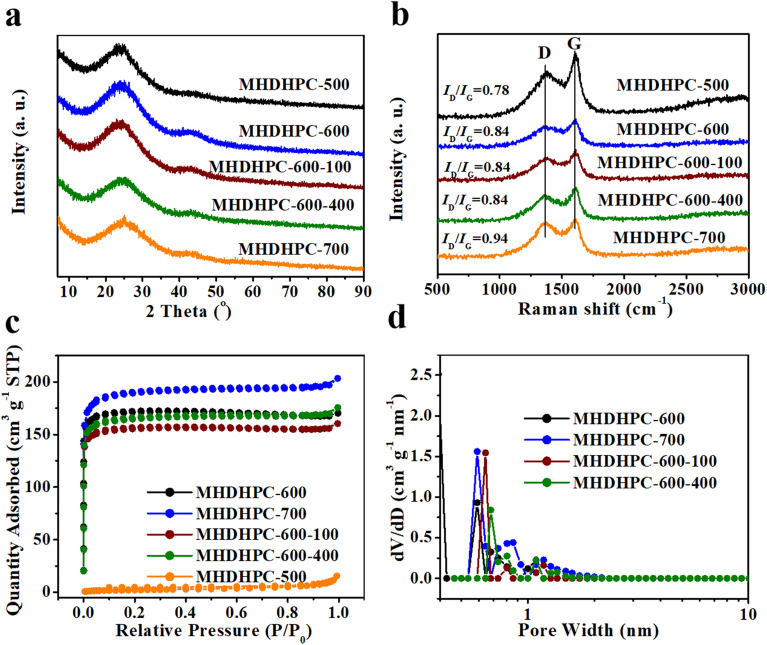
(a) XRD patterns, (b) Raman spectra and (c) N_2_ sorption isotherms of MHDHPC-500, MHDHPC-600, MHDHPC-700, MHDHPC-600-100 and MHDHPC-600-400; (d) pore-size distribution of MHDHPC-600, MHDHPC-700, MHDHPC-600-100 and MHDHPC-600-400.

The crystalline structures of MHDHPC-500, MHDHPC-600, MHDHPC-700, MHDHPC-600-100 and MHDHPC-600-400 were characterized using XRD and Raman techniques. Two broad and weak diffraction peaks at 24° and 43° were observed in the XRD patterns of MHDHPC-500, MHDHPC-600, MHDHPC-700, MHDHPC-600-100 and MHDHPC-600-400 (Fig. 3a), indicating the amorphous nature of MHDHPC. The amorphous nature of MHDHPC-500, MHDHPC-600, MHDHPC-700, MHDHPC-600-100 and MHDHPC-600-400 was determined using Raman spectra. Fig. 3b shows Raman spectra displaying two sharp and strong peaks at 1340 cm^−1^ (D-band corresponding to the disordered structures) and 1586 cm^−1^ (G-band corresponding to sp^2^-hybridized graphitic carbon). The high peak intensity of the D-band indicated the primarily disordered structures in MHDHPC-500, MHDHPC-600, MHDHPC-700, MHDHPC-600-100 and MHDHPC-600-400. The intensity ratio (*I*_D_/*I*_G_) between the D-band and G-band is usually used to determine the graphitization degrees of carbon materials. The value of *I*_D_/*I*_G_ increased from 0.78 for MHDHPC-500 to 0.94 for MHDHPC-700. MHDHPC-700 had the lowest graphitization degree, due to the increased amount of nanopores as a result of the stronger activation process with KOH that introduced structural defects to the carbon matrix. It should be mentioned that the identical *I*_D_/*I*_G_ values of MHDHPC-600, MHDHPC-600-100 and MHDHPC-600-400 illustrated the same graphitization degree.

The porous structures of MHDHPC-500, MHDHPC-600, MHDHPC-700, MHDHPC-600-100 and MHDHPC-600-400 were characterized using nitrogen physisorption measurements in liquid nitrogen. A very sharp and steep adsorption feature below 0.1*P*/*P*_0_ and inconspicuous hysteresis loops above 0.1*P*/*P*_0_ are both shown in the isotherms (Fig. 3c) of MHDHPC-600, MHDHPC-700, MHDHPC-600-100 and MHDHPC-600-400, revealing the predominantly microporous structures of all MHDHPC samples. A slight uptake above 0.9*P*/*P*_0_ in the isotherms of MHDHPC-600, MHDHPC-700, MHDHPC-600-100 and MHDHPC-600-400 indicated the existence of some macropores in all MHDHPC samples, which was in accordance with the SEM results. However, there was no obvious adsorption for MHDHPC-500 at all pressures, indicating that the KOH thermal activation reaction with carbon did not occur. Fig. 3d shows pore-size distribution plots of MHDHPC-600, MHDHPC-700, MHDHPC-600-100 and MHDHPC-600-400, displaying microporous structures, which are very different from MHDHPC-500 showing mesoporous and macroporous structures (Fig. S7). The porous features of MHDHPC-500, MHDHPC-600, MHDHPC-700, MHDHPC-600-100 and MHDHPC-600-400 are better illustrated by the pore structural parameters listed in Table 1. MHDHPC-600 MHDHPC-600-100, MHDHPC-600-400 and MHDHPC-700 had extremely high micropore proportions both in the SSA and in the total pore volume, revealing the microporous features of all MHDHPC samples. It should be noted that the BET SSAs of MHDHPC-600 and MHDHPC-700 were much higher than that of MHDHPC-500, demonstrating the formation of numerous microporous structures in MHDHPC-600 and MHDHPC-700. The microporous SSA and pore volume of MHDHPC-700 were higher than those of MHDHPC-600, indicating that the microporous structures could be generated by increasing the carbonization temperature. Meanwhile, the mass density of MHDHPC-700 (1.25 g cm^−3^) was lower than that of MHDHPC-600 (1.33 g cm^−3^), which was unfavorable for obtaining high volumetric capacitance performance in supercapacitors. More importantly, MHDHPC-600-100 and MHDHPC-600-400 both had similar porous structures and mass densities to MHDHPC-600. Combined with the same morphological structure and the same graphitization degree, this made MHDHPC-600 suitable for large-scale production.

**Table 1:** Pore structural parameters of MHDHPC-500, MHDHPC-600, MHDHPC-700, MHDHPC-600-100 and MHDHPC-600-400

Sample	*S*_BET_ (m^2^ g^−1^)	*S*_micro_ (m^2^ g^−1^)	*V*_total_ (cm^3^ g^−1^)	*V*_micro_ (cm^3^ g^−1^)	*ρ* (g cm^−3^)
MHDHPC-600	501	448	0.25	0.24	1.33
MHDHPC-600-100	456	403	0.24	0.21	1.35
MHDHPC-600-400	485	474	0.26	0.25	1.31
MHDHPC-500	8	0.089	0.02	0.00	1.92
MHDHPC-700	559	543	0.30	0.29	1.25

**Fig. 4:**
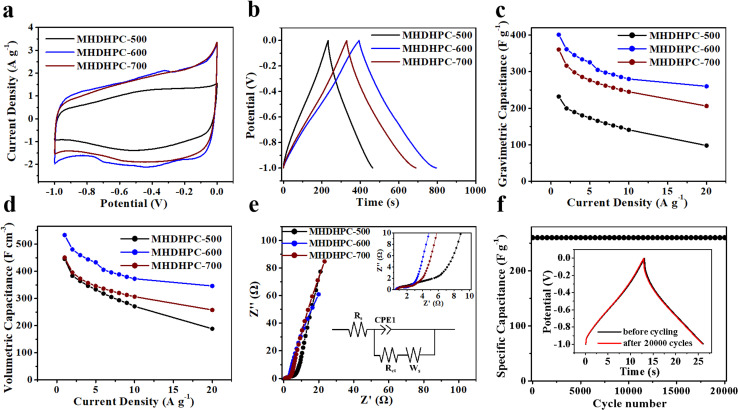
(a) CV curves at 5 mV s^−1^, (b) GCD curves at 1 A g^−1^, (c) *C*_g_ and (d) *C*_v_ derived from GCD curves, and (e) Nyquist plots of MHDHPC-500, MHDHPC-600 and MHDHPC-700; (f) stability evaluation of MHDHPC-600.

The electrochemical properties of MHDHPC-500, MHDHPC-600 and MHDHPC-700 were measured in a three-electrode system in 6 M KOH. Fig. 4a shows cyclic voltammetry (CV) curves of MHDHPC-500, MHDHPC-600 and MHDHPC-700 measured at 5 mV s^−1^, and all CV curves exhibit nearly rectangular shapes, revealing the predominant electric double-layer capacitance (EDLC) features. The largest internal area of the CV curve in MHDHPC-600 implied that MHDHPC-600 had a higher specific capacitance than MHDHPC-500 and MHDHPC-700. The rectangular shapes of all the CV curves were well retained, even at 200 mV s^−1^ (Fig. S8). It should be mentioned that the CV curve of MHDHPC-600 (Fig. 4a) exhibited a broad redox peak at a wide potential range of −0.2 to −0.8 V, due to the overlap of complex redox reactions derived from the N and S groups (eqn (1) and (2)).^35,36^

1 –C–N–C– + H_2_O + e^−^ ↔ –C–NH–C– + OH^−^

2 –C–S–C– + H_2_O + 3e^−^ ↔ –C–SO_2_–C– + 3OH^−^

Fig. 4b shows galvanostatic charge discharge (GCD) curves of MHDHPC-500, MHDHPC-600 and MHDHPC-700 measured at 1 A g^−1^, and all GCD curves exhibited ideal triangular shapes, revealing good EDLC features. The longest charge/discharge time of the GCD curve for MHDHPC-600 further demonstrates its superior capacitance compared to MHDHPC-500 and MHDHPC-700. The gravimetric specific capacitances (*C*_g_) and volumetric specific capacitances (*C*_v_) are shown in Fig. 4c and d, respectively. MHDHPC-600 had both the highest *C*_g_ and *C*_v_ at all current densities compared to MHDHPC-500 and MHDHPC-700, which may be due to the synergistic effect of the honeycomb-like macroporous network structure with predominantly microporous structures leading to the significant generation of double-layer capacitance and surface heteroatom-rich nature (8.8 at% for O, 2.1 at% for N and 1.3 at% at for S), resulting in a large pseudocapacitance being obtained. The *C*_g_ (400.9 F g^−1^) of MHDHPC-600 was superior to those of other advanced HPC materials derived from cauliflower (311 F g^−1^),^20^ grape (275 F g^−1^),^21^ lignin (348 F g^−1^),^22^ wheat bran (294 F g^−1^),^24^ waste cottonseed husks (238 F g^−1^),^25^*Ganoderma lucidum* spores (224 F g^−1^),^26^ and cellulose pulp (210 F g^−1^),^41^ and comparable to those of garden cress seed-derived HPC (409 F g^−1^),^27^ instant dry yeast derived HPC (492 F g^−1^),^28^ and sewage sludge derived HPC (492 F g^−1^),^46^ as well as higher than those of other reported porous carbon materials shown in Table 2. Because of its very high density and high *C*_g_, *C*_v_ of MHDHPC-600 reached up to 533 F cm^−3^ at 1 A g^−1^, which was superior to previously reported novel porous carbon in an aqueous electrolyte (Table 2). The *C*_g_ and *C*_v_ of MHDHPC-600 at 1 A g^−1^ were much higher than the values of 278 F g^−1^ and 216 F cm^−3^ of commercial activated carbon at 1 A g^−1^ in 6 M KOH (Fig. S9a). At 20 A g^−1^, *C*_v_ of MHDHPC-600 remained at 345 F cm^−3^, which was much higher than those of MHDHPC-500 (188 F cm^−3^) and MHDHPC-700 (227 F cm^−3^), as well as higher than those for other reported novel porous carbon materials shown in Table 2. More importantly, the *C*_g_ and *C*_v_ of MHDHPC-600 were as high as 299 F g^−1^ and 397 F cm^−3^ at 0.1 A g^−1^ with an active material loading of 10 mg per cm^2^ per electrode (Fig. S9b). The electrochemical impedance spectroscopy (EIS) curves of MHDHPC-500, MHDHPC-600 and MHDHPC-700 were measured in the frequency range from 0.01 to 100 000 Hz with a perturbation amplitude of 5 mV. As shown in Fig. 4e, nearly vertical lines appeared in the EIS curves of MHDHPC-500, MHDHPC-600 and MHDHPC-700, indicating their fast ion diffusion behavior and good capacitive performance. Compared to MHDHPC-500 and MHDHPC-700, MHDHPC-600 exhibited the lowest equivalent series resistance of 0.55 Ω, obtained using the *x*-intercept of the EIS curve, revealing better conductivity of MHDHPC-600. The semicircle diameter of MHDHPC-600 in the high-frequency region was smaller than those of MHDHPC-500 and MHDHPC-700, indicating its lower charge-transfer resistance. The cycling stability of MHDHPC-600 was measured by GCD testing at 20 A g^−1^. MHDHPC-600 exhibited excellent electrochemical cycling stability of 100% after 20 000 cycles (Fig. 4f), indicating long-term stability. The chemical composition of the MHDHPC-600 electrode after 20 000 cycles was analyzed using X-ray photoelectron spectroscopy. As shown in Fig. S10, N and S heteroatoms still survived in MHDHPC-600, indicating its very good cycle stability.

**Table 2:** Comparison of the *C*_v_ and *C*_g_ of previously reported porous carbon materials in aqueous electrolytes

Material	BET SSA (m^2^ g^−1^)	Pore volume (cm^3^ g^−1^)	*C*_v_/*C*_g_	*C*_v_	Ref.
Fungus-derived carbon	1103	0.54	360 F cm^−3^/374 F g^−1^ at 0.5 A g^−1^	—	29
Soybean-derived porous carbon	580	—	468 F cm^−3^/425 F g^−1^ at 0.5 A g^−1^	341 F cm^−3^ at 20 A g^−1^	30
Honeycomb-like porous carbon	832	—	349 F cm^−3^ at 0.1 A g^−1^	200 F cm^−3^ at 20 A g^−1^	31
Elm samara-derived porous carbon nanosheet	1947	1.33	257 F cm^−3^/470 F g^−1^ at 1.0 A g^−1^	145 F cm^−3^ at 20 A g^−1^	32
Porous carbon nanosheet	539	0.4	317 F cm^−3^/286 F g^−1^ at 0.5 A g^−1^	—	47
Porous carbon	387	0.29	355 F cm^−3^/309 F g^−1^ at 0.5 A g^−1^	255 F cm^−3^ at 10 A g^−1^	48
*Perilla frutescens*-derived porous carbon nanosheets	655	0.44	287 F cm^−3^/270 F g^−1^ at 0.5 A g^−1^	216 F cm^−3^ at 20 A g^−1^	49
Porous carbon spheres	503	0.78	219 F cm^−3^/274 F g^−1^ at 0.5 A g^−1^	—	50
Phosphorus- and nitrogen-enriched porous carbons	16.1	—	261 F cm^−3^/205.7 F g^−1^ at 0.5 A g^−1^	—	51
Microporous carbon spheres	510.6	0.25	170 F cm^−3^/164 F g^−1^ at 1.0 A g^−1^	138 F cm^−3^ at 20 A g^−1^	52
Porous carbon spheres	1412.9	0.68	257.7 F cm^−3^/378.9 F g^−1^ at 0.05 A g^−1^	213.2 F cm^−3^ at 20 A g^−1^	53
MHDHPC-600	501	0.25	533 F cm^−3^/400.9 F g^−1^ at 1.0 A g^−1^	345 F cm^−3^ at 20 A g^−1^	This work

**Fig. 5:**
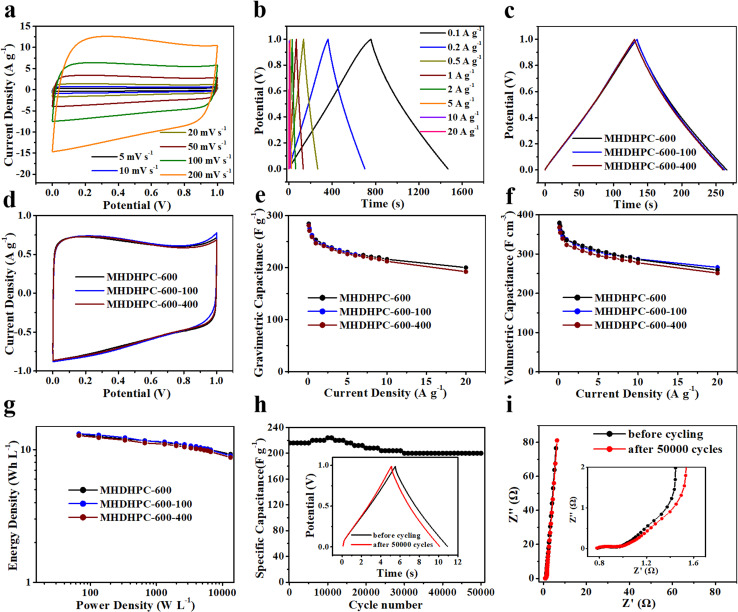
Electrochemical performance of MHDHPC-600, MHDHPC-600-100 and MHDHPC-600-400 based symmetrical supercapacitors in 6 M KOH. (a) CV curves and (b) GCD curves of MHDHPC-600; (c) GCD curves at 0.5 A g^−1^, (d) CV curves at 10 mV s^−1^, (e) *C*_g_, (f) *C*_v_ and (g) Ragone plots of MHDHPC-600, MHDHPC-600-100 and MHDHPC-600-400; (h) cycling stability at 10 A g^−1^ and (i) corresponding Nyquist plots of MHDHPC-600.

Due to the superior electrochemical performance of MHDHPC-600, scalable production experiments were carried out and symmetric supercapacitor devices were assembled in 6 M KOH. Fig. 5a shows CV curves of MHDHPC-600 displaying nearly rectangular shapes even at 200 mV s^−1^, revealing its outstanding EDLC characteristics and excellent rate capacitance. The nearly symmetrical triangle shape in all GCD curves of MHDHPC-600 (Fig. 5b) further indicated outstanding capacitive behavior. Both CV curves and GCD curves of MHDHPC-600-100 and MHDHPC-600-400 (Fig. S11) displayed the same shapes as those of MHDHPC-600, demonstrating excellent reproducibility and reliability of the electrochemical performance of MHDHPC. Significantly, almost overlapping GCD curves at 0.5 A g^−1^ (Fig. 5c)/10 A g^−1^ (Fig. S12a) and CV curves at 10 mV s^−1^ (Fig. 5d)/100 mV s^−1^ (Fig. S12b) demonstrated that MHDHPC-600, MHDHPC-600-100 and MHDHPC-600-400 had nearly identical capacitances, which were confirmed by capacitive performances, as shown in Fig. 5e and f. At 0.1 A g^−1^, the *C*_g_ (*C*_v_) values of MHDHPC-600, MHDHPC-600-100 and MHDHPC-600-400 were 284 F g^−1^ (377 F cm^−3^), 281 F g^−1^ (379 F cm^−3^) and 280 F g^−1^ (366 F cm^−3^), respectively. MHDHPC-600 exhibited a good capacitance retention of 70% as the current density increased to 20 A g^−1^. Ragone plots of MHDHPC-600, MHDHPC-600-100 and MHDHPC-600-400 are shown in Fig. 5g and S13, which show almost overlapping Ragone plots, demonstrating that MHDHPC-600, MHDHPC-600-100 and MHDHPC-600-400 had nearly identical energy densities. The *E*_v_ (*E*_g_) values of MHDHPC-600, MHDHPC-600-100 and MHDHPC-600-400 were 13.1 Wh L^−1^ (9.87 Wh kg^−1^), 13.1 Wh L^−1^ (9.76 Wh kg^−1^), and 12.7 Wh L^−1^ (9.75 Wh kg^−1^) at 66.5 W L^−1^ (50 W kg^−1^), respectively. Significantly, the *E*_v_ of 13.1 Wh L^−1^ was higher than those of most reported carbon-based supercapacitors in 6 M KOH, such as garden cress seed-derived HPC (6.2 Wh L^−1^),^27^ strutted porous carbon nanosheets (6.5 Wh L^−1^),^47^ nitrogen-doped active carbon/graphene (11.1 Wh L^−1^),^53^ porous carbon nanosheets (9.2 Wh L^−1^),^54^ graphene films (2.78 Wh L^−1^),^55^ nitrogen-enriched lignin porous carbon (5.1 Wh L^−1^),^56^ dense porous carbon (8.26 Wh L^−1^),^57^ and N-enriched functional carbon (13.01 Wh L^−1^).^58^ The superior *C*_g_, *C*_v_, *E*_v_ and *E*_g_ and high rate capability of MHDHPC-600 may be attributed to the honeycomb-like macroporous network structure, high SSA with micropore-dominance and high content of N/S heteroatoms. More importantly, the MHDHPC-600-based symmetric supercapacitor exhibited ultrahigh cycling stability of 92% after 50 000 cycles at 10 A g^−1^ (Fig. 5h). The *C*_g_ of 200 F g^−1^ after 50 000 cycles represented slight degeneration compared to the initial *C*_g_ value (216 F g^−1^). Fig. 5i shows the EIS curves before/after 50 000 cycles, displaying almost vertical lines, revealing excellent capacitive behavior. As shown in the inset figure in Fig. 5i, a very small semicircle diameter was observed in the low frequency region, indicating that MHDHPC-600 exhibited a very low charge-transfer resistance and fast ion diffusion behavior. The almost overlapping EIS curves before/after 50 000 cycles suggested that MHDHPC-600 had outstanding cycling stability. This new generation of MHDHPC represents an important breakthrough for improving the gravimetric/volumetric energy density of supercapacitors with high power and outstanding cycling stability. The good gravimetric/volumetric electrochemical performance and easily scalable manufacture at low cost suggest that MHDHPC-600 has excellent potential applications in the supercapacitor industry.

The superior electrochemical performance of MHDHPC-600 in 6 mol L^−1^ KOH could be due to the following reasons: (i) honeycomb-like interconnected macropores provide pathways for electrolyte ions, while the abundant micropores provide numerous adsorption sites, enhancing double-layer capacitance. (ii) The high specific surface areas with interconnected hierarchical porous structures ensure a highly accessible surface area for ion storage and diffusion. (iii) A structure rich in oxygen-/nitrogen-/sulfur-containing groups not only improves the wettability of the carbon materials but also contributes to the generation of extra pseudocapacitance. (iv) The relatively high mass density and high gravimetric specific capacitance could ensure high volumetric specific capacitances.

## Conclusions

In conclusion, we report a facile, reproducible and scalable synthesis of multiple heteroatom-doped honeycomb-like porous carbon (MHDHPC) from cotton pulp board. MHDHPC exhibits ultrahigh volumetric capacitance and volumetric energy density, high rate capability and excellent cycling stability, due to its high surface area, ultrahigh microporosity, high density and surface heteroatom-rich nature. Even after scaling up the experiments sixteen times, the surface morphology, porous strcuture, and electrochemical performance remained well preserved. Most importantly, we demonstrate that this synthetic strategy provides high stability, excellent reproducibility and high yield, and can be employed in the scalable synthesis of MHDHPC. Therefore, this work provides a promising strategy to employ low-cost biomass for the scalable synthesis of advanced porous carbon materials for high volumetric-performance supercapacitors.

## Author contributions

C. Chen and P. H. Zhang designed the experiments. W. X. Zhong carried out electrochemical performance tests. Y. X. Su and K. Y. Zhang provided characterization *via* SEM, TEM, XRD, XPS, Raman and N_2_ adsorption–desorption tests. C. Chen and P. H. Zhang wrote the paper.

## Conflicts of interest

The authors declare no competing interests.

## Supplementary Material

RA-016-D5RA08165C-s001

## Data Availability

The data supporting this article have been included as part of the supplementary information (SI). Supplementary information is available. See DOI: https://doi.org/10.1039/d5ra08165c.
