# Supplementary material for: Scalable synthesis of N,S co-doped honeycomb-like porous carbon with micropore-dominance for ultrahigh volumetric-performance supercapacitors
Source: RSC Adv. 2026 Jan 12;16(3):2610–20. doi: 10.1039/d5ra08165c (PMC12794195; doi:10.1039/d5ra08165c)
Supplement: RA-016-D5RA08165C-s001 [file RA-016-D5RA08165C-s001.pdf]

Supplementary Information (SI) for RSC Advances.
This journal is © The Royal Society of Chemistry 2025

Electronic Supplementary Information

Scalable synthesis of N, S co-doped honeycomb-like porous carbon with a micropore-dominant for ultrahigh volumetric performance supercapacitors

Chong Chen,*[a] Yongxiang Su,[a] Wenxiu Zhong[a],[a] Keying Zhang,[a] and Pinghua Zhang*[a]

[a] Key Laboratory of Spin Electron and Nanomaterials of Anhui Higher Education Institutes, School of Chemistry and Chemical Engineering, Suzhou University, Suzhou 234000, People's Republic of China

* Corresponding author. E-mail addresses: szxychenchong@163.com (C. Chen), zphchemical@163.com (P. H. Zhang)

Materials characterizations

The morphology and microstructure were characterized by scanning electron microscopy (SEM, Hitachi SU8100) and transmission electron microscopy (TEM, Jem-2100F Jeol). Structure of MHDHPC were analyzed by X-ray diffraction (XRD, SmartLab3KW) and Raman spectrometer (Thermofisher Dxr2xi). The chemical composition and states of MHDHPC were analyzed by X-ray photoelectron spectroscopy (XPS, Thermo Escalab 250Xi). Nitrogen adsorption-desorption isotherms of MHDHPC were tested by an ASAP-2020 at 77 K. The pore size distribution plots calculated by density functional theory. The thermogravimetric analyser were measured on Mettler DSC3 under the protection of Ar gas with a ramping rate of 10 °C min^{-1}.

Electrochemical measurements

The electrochemical performances of MHDHPC were evaluated by using electrochemical workstation (ChenHua, CHI760D) in 6 M KOH via cyclic voltammetry (CV), galvanostatic charge-discharge (GCD) and electrochemical impedance spectra (EIS) measurements. To prepare the MHDHPC electrodes, active materials (80 wt%), acetylene black (15 wt%) and polytetrafluoroethylene binder (5 wt%) were mixed together using ethanol to make a slurry, which was coated onto nickel foam sheets with uniform thickness (1.0 cm × 1.0 cm) followed by drying at 100 °C for 12 h. The loading weight of the active materials in MHDHPC electrode was about 1.5 mg cm^{-2}. The three-electrode systems were composed of working electrode, Pt foil counter electrode and Hg/HgO reference electrode. For the two-electrode system, the symmetric supercapacitor was assembled by two identical MHDHPC electrodes as a 2025 stainless steel coin cell, using glass fiber as the separator.

The gravimetric specific capacitances C_g (F g^{-1}) respective in the three-electrode systems (equation 1) and in the two-electrode systems (equation 2) were calculated from GCD curves accorrding to the follow equation :

$$C_{g} = \frac{I\Delta t}{m\Delta V} \tag{1}$$

$$C_{g} = \frac{4I\Delta t}{m\Delta V} \tag{2}$$

where I (A) is the discharge current; Δt (s) is the discharge time; ΔV (V) is the potential window; and m (g) is the mass of MHDHPC.

The volumetric specific capacitances C_{v} (F cm^{-3}) both in the three-electrode systems and in the two-electrode systems were calculated based on the following equation:

$$C_{v} = C_{g} \times \rho \tag{3}$$

$$\rho = \frac{1}{V_{total} + \frac{1}{\rho_{carbon}}} \tag{4}$$

where ρ (g cm^{-3}) is the density of MHDHPC materials; V_{total} (cm^{3} g^{-1}) is the total pore volume of MHDHPC; and ρ_{carbon} is the true density of carbon (2 g cm^{-3}).

Energy density E_{g} (Wh kg^{-1}) / E_{v} (Wh L^{-1}) and power density P_{g} (W kg^{-1}) / P_{v} (W L^{-1}) of the symmetric supercapacitors were calculated from the equations:

$$E_{g} = \frac{1}{8} C_{g} \Delta V^{2} \tag{5}$$

$$P_{g} = \frac{E_{g}}{\Delta t} \tag{6}$$

$$E_{v} = \frac{1}{8} C_{v} \Delta V^{2} \tag{7}$$

$$P_{v} = \frac{E_{v}}{\Delta t} \tag{8}$$

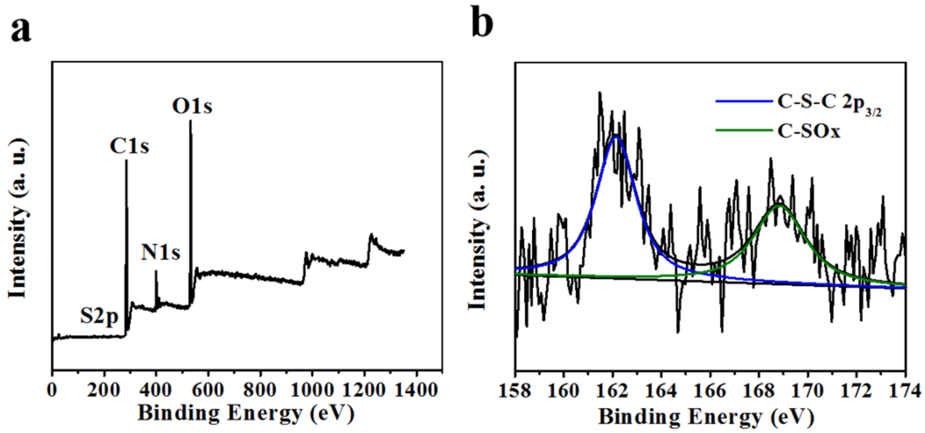

Fig. S1 (a) XPS survey and (b) S2p spectra of cotton pulp board.

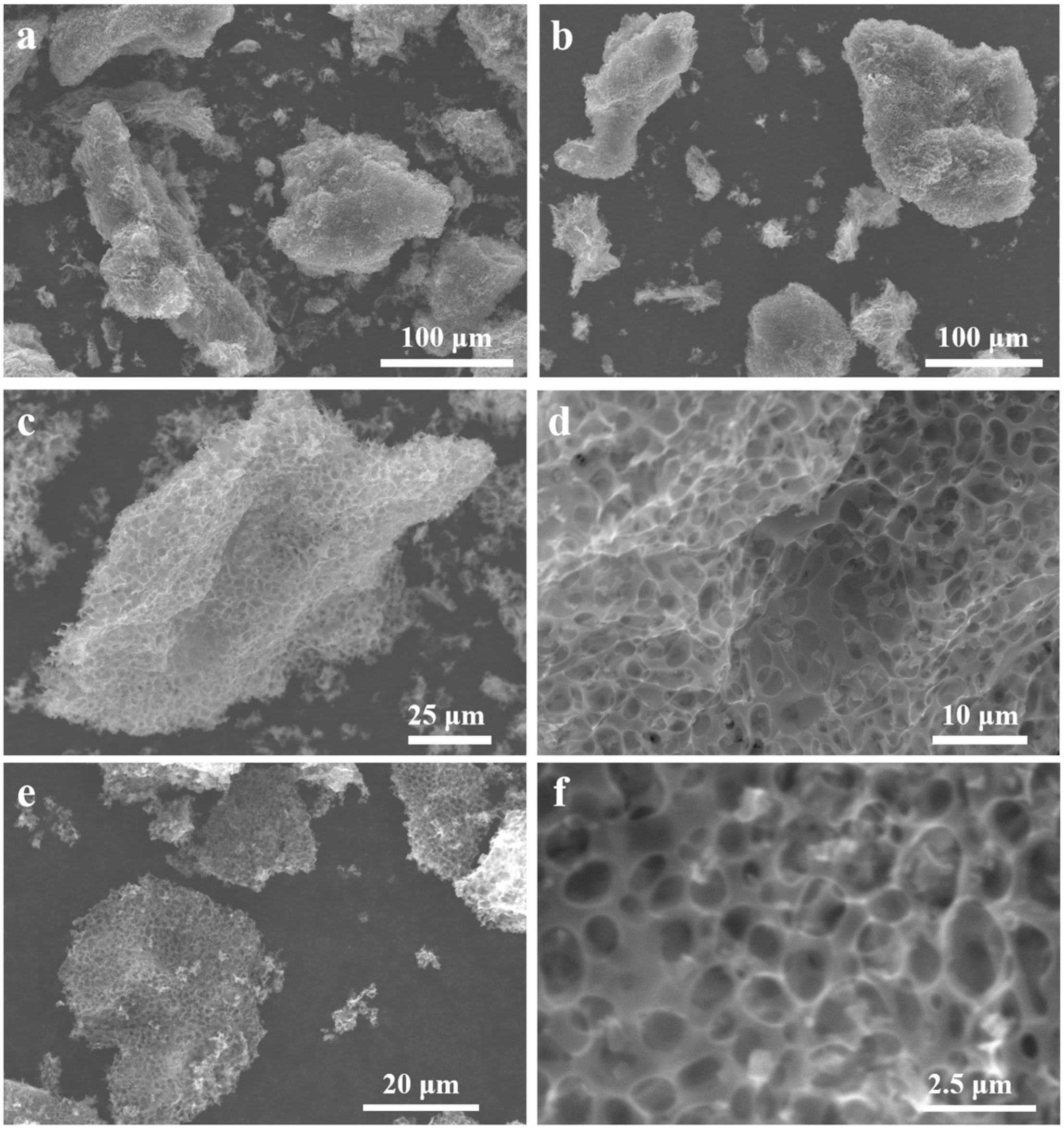

Fig. S2 Low-magnification SEM images of (a) MHDHPC-600-100 and (b) MHDHPC-600-400; (c) Low-magnification and (d) high-magnification SEM images of MHDHPC-500; (e) Low-magnification and (f) high-magnification SEM images of MHDHPC-700.

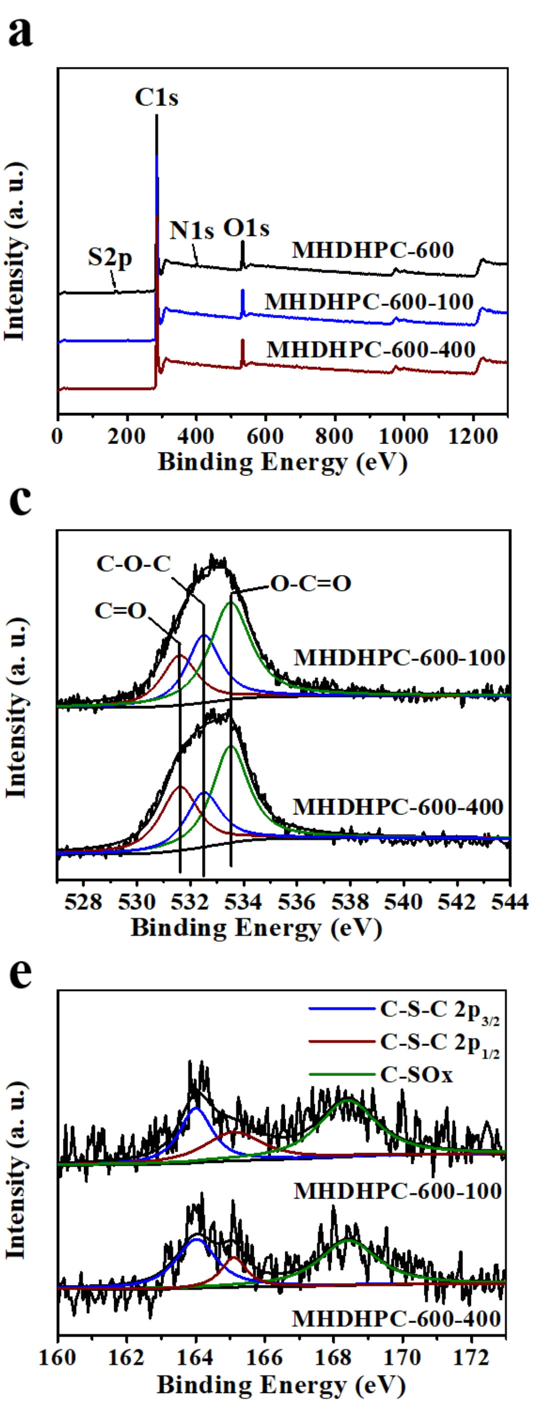

Fig. S3 (a) XPS surveys of and MHDHPC-600, MHDHPC-600-100 and MHDHPC-600-400; (b) C1s, (c) O1s, (d) N1s and (e) S2p spectra of MHDHPC-600-100 and MHDHPC-600-400.

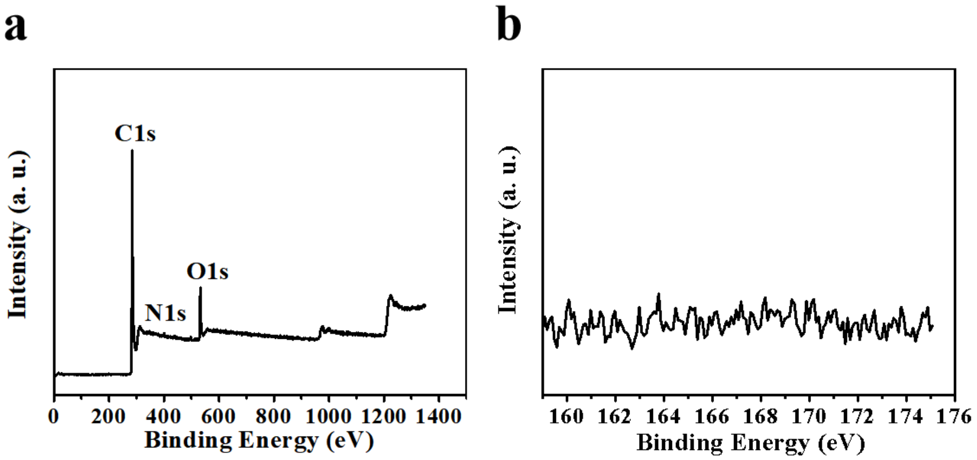

Fig. S4 (a) XPS surveys and (b) S2p spectra of MHDHPC-700.

Table S1 XPS results of MHDHPC-600, MHDHPC-600-100, MHDHPC-600-400, MHDHPC-700 and cotton pulp.

Sample	C (at.%)	N (at.%)	O (at.%)	S (at.%)
MHDHPC-600	87.8	2.1	8.8	1.3
MHDHPC-600-100	89.7	1.1	8.8	0.4
MHDHPC-600-400	88.8	1.7	9.1	0.4
MHDHPC-700	88.6	1.3	10.4	-
cotton pulp	67.0	8.8	23.6	0.6

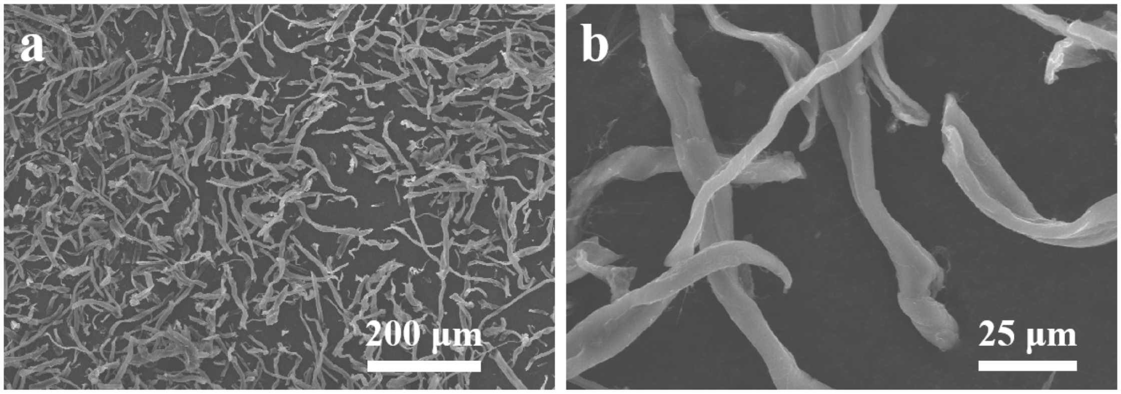

Fig. S5 SEM images of KOH-CP0.

Table S2 Pore structural parameters of KOH-CP0.5, MHDHPC-600 and KOH-CP1.5.

Sample	S_{BET} ($m^2\ g^{-1}$)	S_{micro} ($m^2\ g^{-1}$)	V_{total} ($cm^3\ g^{-1}$)	V_{micro} ($cm^3\ g^{-1}$)	ρ ($g\ cm^{-3}$)
KOH-CP0.5	392	350	0.20	0.19	1.44
MHDHPC-600	501	448	0.25	0.24	1.33
KOH-CP1.5	434	381	0.223	0.200	1.38

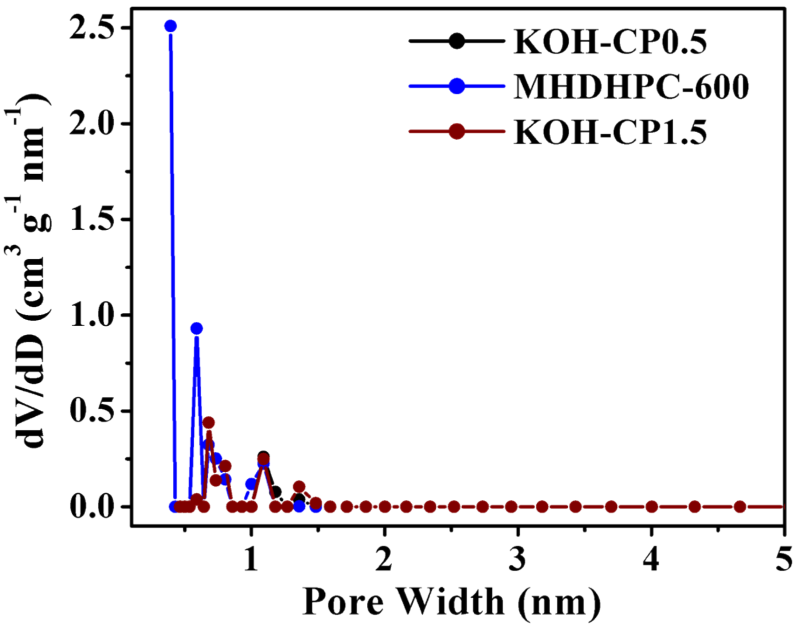

Fig. S6 Pore size distribution plots of KOH-CP0.5, MHDHPC-600 and KOH-CP1.5.

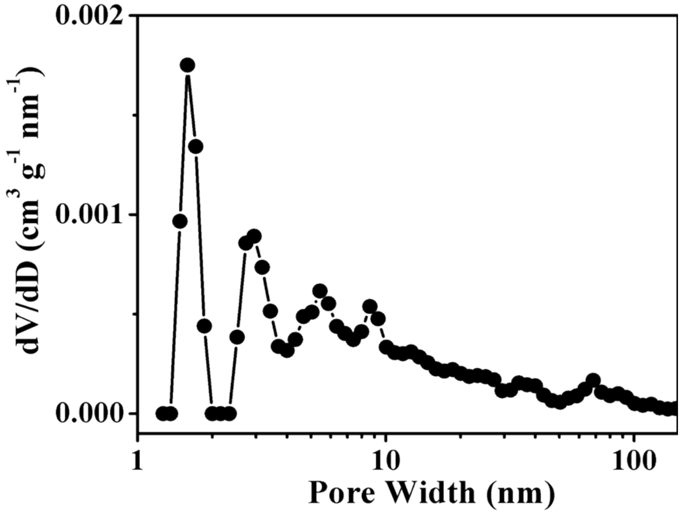

Fig. S7 Pore-size distribution of MHDHPC-500.

Fig. S8 CV curves of MHDHPC-600 in a three electrode system in 6M KOH.

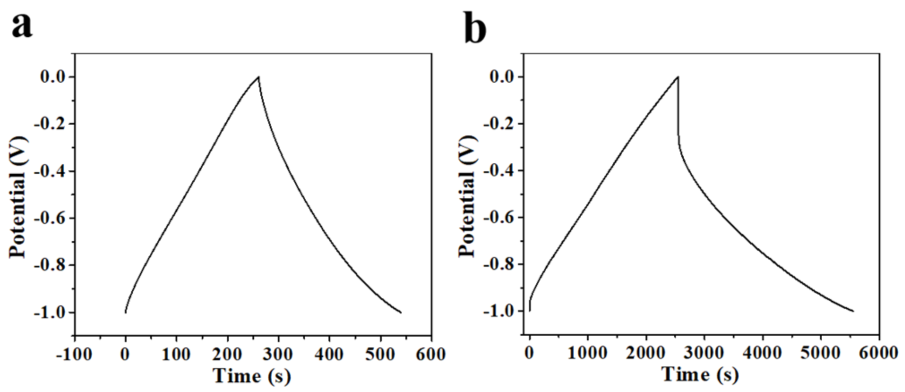

Fig. S9 (a) GCD curve of commercial activated carbon at 1 A g^{-1} in a three electrode system in 6M KOH with an active material loading of 1.5 mg cm^{-2} per electrode; (b) GCD curve of MHDHPC-600 at 0.1 A g^{-1} in a three electrode system in 6M KOH with an active material loading of 10 mg cm^{-2} per electrode.

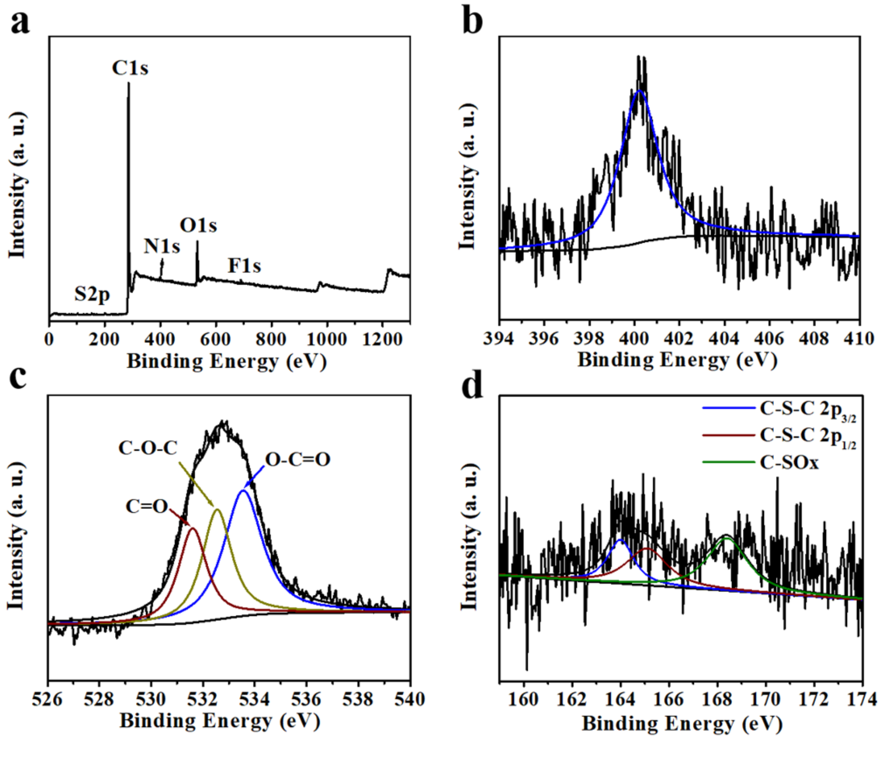

Fig. S10 After 20,000 GCD cycles at 20 A g^{-1} in a three electrode system in 6 M KOH, the MHDHPC-600 electrode was washed by 1 M HCl solution and was followed by filtration with distilled water, which was then dried at 100 ºC for 12 h. (a) XPS survey; (b) N1s; (c) O1s and (d) S2p spectra MHDHPC-600 electrode after 20,000 cycles.

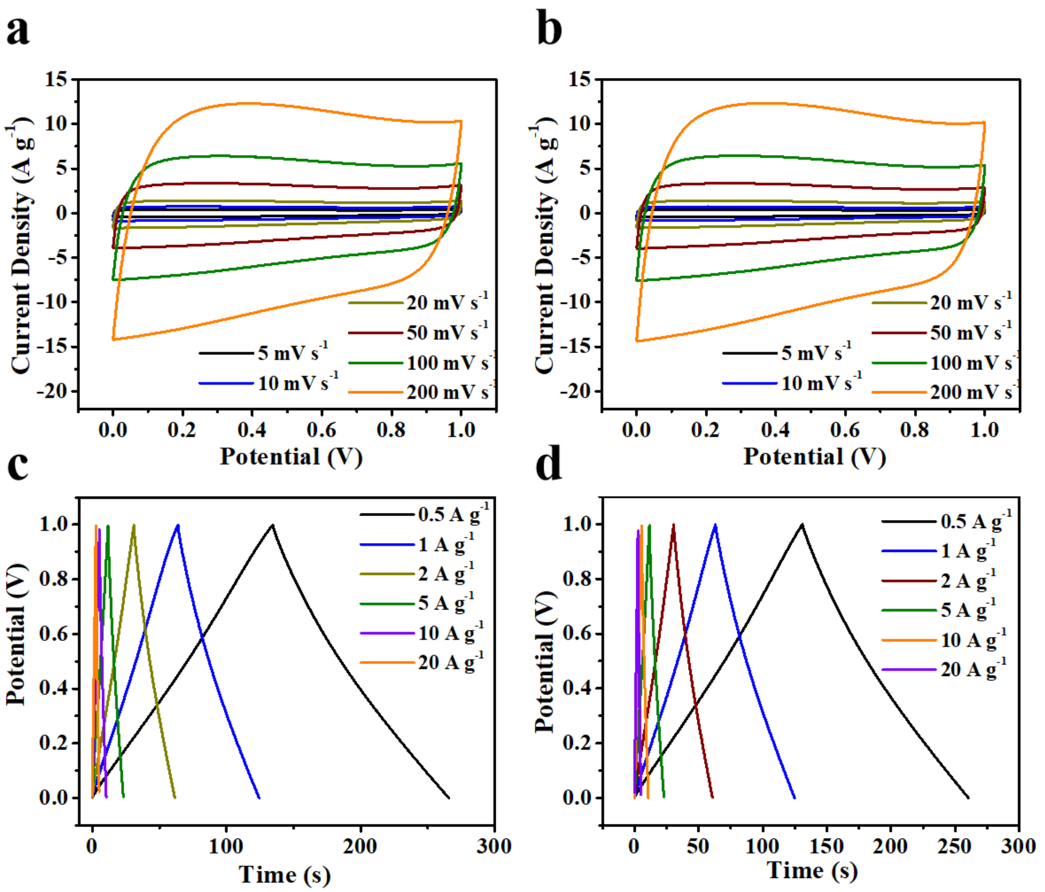

Fig. S11 CV curves of (a) MHDHPC-600-100 and (b) MHDHPC-600-400; GCD curves of (c) MHDHPC-600-100 and (d) MHDHPC-600-400.

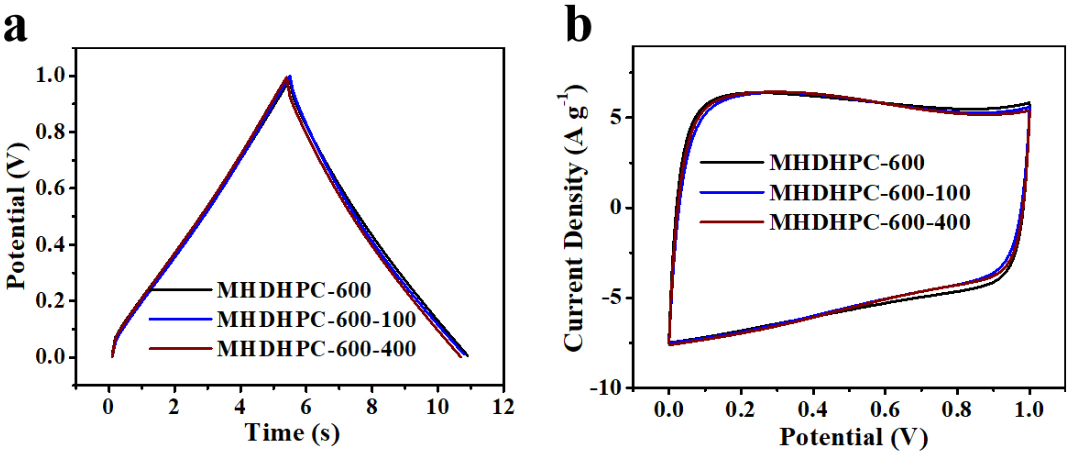

Fig. S12 (a) GCD curves at 10 A g^{-1} and (b) CV curves at 100 mV s^{-1} of MHDHPC-600, MHDHPC-600-100 and (d) MHDHPC-600-400.

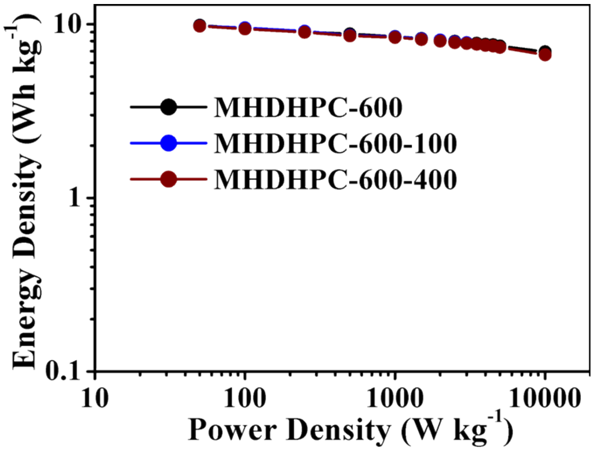

Fig. S13 Ragone plots of MHDHPC-600, MHDHPC-600-100 and MHDHPC-600-400.
